# Comparison of auditory brainstem response and auditory steady state response audiometry by evaluating the hearing thresholds obtained in children with different severity of hearing loss

**DOI:** 10.12669/pjms.35.2.688

**Published:** 2019

**Authors:** Muhammad Azeem Aslam, Adeela Javed, Abdul Moiz

**Affiliations:** 1 *Muhammad Azeem Aslam, MBBS, FCPS, M.Phil. (Hearing Sciences), Professor and Head, Department of ENT, Al-Nafees Medical College, Isra University, Islamabad Campus, Pakistan*; 2 *Adeela Javed, M.Phil (Hearing Sciences), Audiologist, Department of ENT, Al-Nafees Medical College, Isra University, Islamabad Campus, Pakistan*; 3 *Abdul Moiz, B.S. Audiology, Audiologist, Department of ENT, Al-Nafees Medical College, Isra University, Islamabad Campus, Pakistan*

**Keywords:** Auditory threshold, Deafness, Evoked potentials, Hearing, Auditory brainstem response, Auditory steady state response

## Abstract

**Objectives:**

To compare the hearing thresholds obtained with auditory brainstem response (ABR) and auditory steady state response (ASSR) audiometry in children with hearing loss.

**Methods:**

Hearing thresholds were obtained by ABR and ASSR in children who presented with suspicion of deafness at Ear, nose & throat department of Al-Nafees Medical College Hospital Islamabad, between January to August 2018. The mean hearing thresholds obtained by two tests were compared within each category of severity of deafness. Time taken by both tests was also compared.

**Results:**

A total of 57 patients (114 ears) were included in the study. Among them 27 (47.4%) were male and 30 (52.6%) were female. The mean age of patients at presentation was 42 months (±30.9) with age range from one to 12 years. Mean hearing thresholds obtained by click ABR, chirp ABR, ASSR (1, 2, 4 kHz) & ASSR (0.5, 1, 2, 4 kHz) was 56.25 (±27.61), 58.88 (±27.44), 58.03 (±21.26) & 56.35 (±22.86) respectively. Mean thresholds were comparable between click ABR & ASSR (1, 2, 4 kHz) and between chirp ABR & ASSR (0.5, 1, 2, 4 kHz) in all degrees of hearing loss categories except in those patients with normal hearing thresholds. The mean time taken by clicks ABR, chirp ABR and ASSR were four minutes seven seconds, three minutes 15 seconds and 16 minutes and 7 seconds respectively.

**Conclusions:**

Hearing thresholds obtained by ABR and ASSR are comparable in all categories of severity of hearing loss. The time taken by ABR is less as compared to ASSR.

## INTRODUCTION

Early identification of hearing loss in infants and children is of utmost importance because normal hearing is essential for speech and intellectual development.^1^ Various age specific subjective and objective audiological tests are being used to evaluate hearing status in children. Among the objective tests, the most commonly used test for this purpose is auditory brainstem response (ABR).^2^ Although ABR is well established and time tested objective audiological test for early identification of hearing loss but it provides limited frequency specific information.^3^ Early auditory rehabilitation whether by modern hearing aids or by cochlear implants depends strongly on frequency specific hearing threshold information.^4^ To overcome this limitation of ABR, a relatively new audiological investigation is gaining popularity in clinical audiological practice which can provide detailed frequency specific thresholds information. This objective audiological test is called auditory steady state responses (ASSR).

After the introduction of ASSR in clinical audiological practice, attempts have been made to compare its various aspects with ABR, a test which is already well established clinically. International literature provides few studies that compared the two techniques but no work has yet been done on this subject in our country. The purpose of this study was to compare hearing thresholds obtained by these two objective audiological tests in children with varying severity of hearing loss and to compare time taken by each test.

## METHODS

This study included all those children from birth till 12 years of age regardless of gender, who due to suspicion of hearing loss, were referred for hearing evaluation to the Audiology section of Department of Otolaryngology, Al-Nafees Medical College Hospital Islamabad, between January to August, 2018. Non-probability convenient sampling technique was used. After taking targeted history from the parents, child’s ear was examined by performing otoscopy. Tympanometry was performed to rule out any conductive cause of hearing loss. Those children who had any congenital anomaly of ear or were suffering from active external and middle ear disease were excluded from the study.

Hearing evaluation was done by ABR & ASSR under natural sleep or sedation (by chloral hydrate) in a purpose build sound treated room. The ambient noise level during the tests was <30 decibels (dB). The equipment used for the test was Sentiero Advanced (PATH Medical, Germany). Supra aural head phones (TD-39) were used to deliver the auditory stimuli to both ears simultaneously. Vertical electrode montage was used & electrode impedance was kept below 5 kohms.

ABR testing was conducted first by click stimuli and then by chirp stimuli using rarefaction polarity. The stimulus rate was 37.1 Hz. Number of averages were 2000. A 10 dB increment or decrement was used to determine the threshold. The upper limit of stimulus intensity was 90 dB HL for click stimuli and 95 dB HL for chirp stimuli. Hearing threshold was defined as the lowest intensity level at which well-defined wave V can be identified by visual inspection.

ASSR was then conducted after the completion of ABR. Adaptive threshold method was used with stimulus intensity range from 10 to 100 dB HL (hearing level). Modulation frequency was 80 Hz. Each ear was tested for four frequencies (0.5, 1, 2 & 4 KHz) simultaneously.

The data obtained was evaluated with statistical package SPSS 21.0 version. Results obtained by both ABR (click & chirp) and ASSR were compared according to the response obtained or not till the maximum upper limit of stimulus intensity. Hearing thresholds obtained by click ABR and ASSR (mean threshold at 1, 2 & 4 kHz) and chirp ABR and ASSR (mean threshold at 0.5, 1, 2 & 4 kHz) were compared within each category of degree of hearing loss. Mean thresholds obtained by click and chirp ABR were also compared. Those cases in which no response was obtained till the upper limit of stimulus presentation of our equipment were not included in the statistical analysis for calculation of mean. Paired sample t test was utilized to compare the means. P value of < 0.01 was interpreted as a statistically significant variation. Pearson correlation test was used to find correlation between the mean thresholds obtained by two techniques. Both the techniques were also evaluated for the mean time taken by each technique.

The study protocol was approved by institutional review board committee of Isra University. Written informed consent was obtained from the parents of each subject. The confidentiality of the participant data was maintained.

## RESULTS

A total of 57 patients (114 ears) were included in the study. Among them, 27 (47.4%) were male and 30 (52.6%) were female. The mean age of patients included in the study was 42 months (3.5 years) SD±30.9 (Range 1 to 12 years). The distribution of patients among various age groups is as follows: 29 in 0-3 years, 18 in 4-6 years, 8 in 7-9 years & only 2 in 10-12 years age groups.

With both click & chirp ABR stimuli, response was observed (i.e., hearing threshold detected) in 43 ears (37.7%) whereas in 71 ears (63.3%) there was no response till the upper limit of stimulus intensity that can be presented with our equipment. With ASSR at four frequencies (i.e., 0.5, 1, 2 & 4 KHz), response was observed in 58 ears (50.9%) whereas in 56 ears (49.1%), no response was recorded on any of the four frequencies up to 100 dB stimulus.

**Fig.1:**
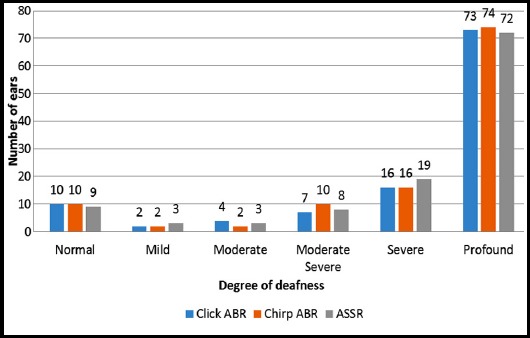
Distribution of patients according to the degree of deafness

The distribution of results according to the degree of deafness with click ABR, chirp ABR & ASSR are shown in Fig.1. In case of ASSR results, mean of four frequency thresholds were utilized to categorize the results according to the severity of deafness.

**Fig.2:**
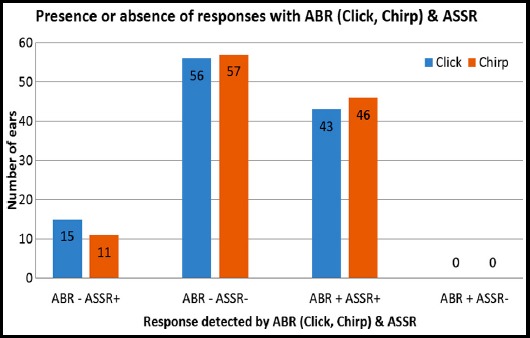
Presence or absence of response with ABR (Click, Chirp) & ASSR.

Both ABR (click & chirp) and ASSR were compared according to the response obtained or not. The results are shown in Fig.2.

None of the ears with no response on ASSR showed response with ABR (either click/chirp) whereas in 15 (13.1%) ears in which click ABR showed no response till the limit of equipment showed response with ASSR and 11 (9.6%) ears in which there was no response with chirp ABR showed response with ASSR. Correlation of mean hearing thresholds obtained with ABR (click and chirp) & ASSR are shown in Table-I:

**Table-I:** Correlation of mean hearing thresholds obtained with ABR & ASSR.

Audiological test	Mean hearing thresholds obtained	Correlation between ABR & ASSR	Pearson’s Correlation	Statistical significance
Click ABR	56.25 (±27.61)	Between Click ABR & ASSR (1,2 & 4 kHz)	0.945	<0.01
Chirp ABR	58.88 (±27.44)			
ASSR (1,2 & 4 kHz)	58.03 (±21.26)	Between Chirp ABR & ASSR (0.5,1,2 & 4 kHz)	0.970	<0.01
ASSR (0.5,1,2& 4 kHz)	56.35 (±22.86)	Between Click & chirp ABR	0.984	<0.01

Hearing thresholds obtained with both the click and chirp ABR were compared with ASSR with in each category of severity of hearing loss. The results are shown in Table-II which suggest that mean hearing thresholds with ABR (both click and chirp) and mean ASSR are comparable (p <0.01) within all degrees of hearing loss except in normal hearing subjects in which ASSR overestimated hearing thresholds as compared to ABR.

**Table-II:** Comparison between mean click & chirp ABR hearing thresholds and mean ASSR thresholds according to degree of hearing loss.

Degree of hearing loss	Mean threshold click ABR	Mean threshold ASSR (1,2 & 4KHz)	p value	Mean threshold Chirp ABR	Mean threshold ASSR (500 Hz, 1,2, & 4 KHz)	p value
Normal	17 (±5.0)	24.2 (±3.72)	<0.01	17 (±5.0)	25 (±4.14)	<0.01
Mild	30 (±0.0)	33.3 (±3.15)	<0.01	30 (±0.0)	31.6 (±2.88)	<0.01
Moderate	50 (±0.0)	53.3 (±3.35)	<0.01	50 (±0.0)	52.5 (±4.33)	<0.01
Moderately Severe	60 (±0.0)	54.9 (±1.96)	<0.01	62.5 (±4.62)	62.8 (±5.03)	<0.01
Severe	82.1(±6.99)	77.2 (±9.45)	<0.01	81.87 (±7.5)	78 (±1.7)	<0.01
Profound	90 (±0.0)	90 (±0.0)	<0.01	91.5 (±2.41)	90 (±0.00)	<0.01

Hearing thresholds obtained by ABR utilizing click and chirp stimuli were also compared with each other. The results are shown in Table-III.

**Table-III:** Comparison of thresholds obtained with chirp and click ABR within each category of deafness.

Severity of hearing loss	Mean threshold by Click ABR	Mean threshold by Chirp ABR	Statistical significance
Normal	17 (±5.0)	17 (±5.0)	<0.01
Mild	30 (±0.0)	30 (±0.0)	<0.01
Moderate	50 (±0.0)	50 (±0.0)	<0.01
Moderately Severe	60 (±0.0)	62.5 (±4.62)	<0.01
Severe	82.14 (±6.99)	81.87 (±7.5)	<0.01
Profound	90 (±0.0)	91.5 (±2.41)	<0.01

Both the techniques were compared with respect to mean time taken by each technique. Mean time taken by click ABR is four minutes and seven seconds and by chirp ABR is three minutes and 15 seconds. Mean time by ASSR test is 16 minutes and seven seconds.

## DISCUSSION

About half of the patients (50.8%) in this study were in age group of one to three years with mean age at the time of presentation for hearing evaluation was 3.5 years which is considerably higher if we compare it with the same in developed countries.^5^ The reason behind late presentation in our country is that there is no hearing screening at birth which is the norm in many developed countries of the world. As no hearing screening is done at birth, many cases of congenital deafness remained undiagnosed^6^ and later on results in either non development or delayed development of speech. This raises the suspicion of deafness due to which these children are referred for hearing evaluation. Another reason for delayed presentation & diagnosis is decreased public awareness of childhood hearing loss and limited availability of specialized audiological equipment and personal. It is well established that earlier the diagnosis of congenital deafness, better will be the results of aural rehabilitation.^7^ The first few years of life is very active learning period and presence of deafness greatly affected the child’s normal cognitive and speech development.^8^ This suggests the need of implementing a mandatory newborn hearing screening program in our country.

ABR and ASSR are the most commonly used objective tests for hearing evaluation in pediatric population. In the present study, hearing thresholds were detected in 37.7% of our patients with ABR utilizing click and chirp stimuli whereas ASSR detected response in about half of the cases (50.9%). One of the reason for this finding is that the upper limit of stimulus presentation with our equipment is 90, 95 and 100 dB for click ABR, chirp ABR and ASSR respectively. There is not a single case in which ABR detected the response but ASSR failed to detect it whereas there were 15 cases tested by click ABR and 11 cases with chirp ABR in which no response was detected but ASSR detected hearing thresholds in all these cases. The possible explanation for these results is that with ASSR technique, higher stimulus levels can be presented which is not possible with ABR. In most auditory evoked potential testing equipment that are clinically available, upper limit of stimulus intensity that can be presented in ABR testing is 90 to 95 dB HL whereas in ASSR, up to 120 dB HL stimulus intensity can be presented. Therefore, ASSR can detect hearing thresholds at a much higher degree of deafness as compared to ABR. This aspect of ASSR is highlighted in many other studies which also suggested that not only the hearing can be tested with higher stimulus intensity but the results of ASSR become more reliable as the severity of hearing loss increases.^5^,^9^

The results of hearing evaluation in our study showed that two thirds of our patients had profound degree of hearing loss. This may be due to the fact that our study included only those children that presented with suspicion of deafness or non-development of speech. This again highlight the need to implement a hearing screening program, as these cases can be diagnosed at birth and appropriate auditory rehabilitation program can be started much earlier.

A high positive correlation (r=0.9) was found when the mean hearing thresholds obtained by click and chirp ABR were compared with ASSR. This finding is in accordance with many other studies in which the two tests were compared.^5^,^9^-^12^ The same correlation was observed when the ABR done by click and chirp stimuli are compared with each other. When mean hearing thresholds obtained with two test techniques were compared (i.e., click ABR with ASSR at 1,2 & 4 kHz and chirp ABR with ASSR at 0.5,1,2 &4kHz), results showed that thresholds obtained with both the test techniques are comparable (p<0.01) at all degrees of hearing loss. Only exception was children in which hearing thresholds were within normal limits. In these cases, ASSR over-estimated the hearing thresholds as compared with ABR. Many earlier studies also mentioned this drawback of ASSR in cases of normal and mild degree of deafness in which ASSR reports over-estimation of hearing threshold.^13^-^16^ Our study did not show any difference of results in mild degree of hearing loss in which both test techniques showed comparable results.

ASSR test took considerable more time to complete the test as compared to ABR but it give frequency specific hearing thresholds information which is not available with click or chirp ABR. This feature is also observed in many other studies.^2^,^17^ When click and chirp ABR tests were compared with respect to the time taken to complete the test, it was found that with chirp ABR, test time was less than with click ABR. This is because chirp stimulus produce wave V of higher amplitude as compared to click stimuli, resulting in better signal to noise ratio thus requiring less time to complete the test. This finding is in accordance with the results of other studies.^18^

## CONCLUSIONS

The hearing thresholds obtained by both the ABR and ASSR are comparable in subjects with varying severity of hearing loss. The time taken to complete the test is much less with ABR than ASSR.

### Author’ Contributions

**MAA:** Conceived and designed the study, analysis and interpretation of data, critically reviewed the manuscript before submission to journal. He also takes responsibility and is accountable for all aspects of the work in ensuring that questions related to the accuracy and integrity of any part of the work are appropriately investigated and resolved.

**AJ & AM:** Both of them did data collection & data analysis. They were also involved in manuscript writing.

**AM:** Did statistical analysis and interpretation of data.
